# Authors’ Response to Letter on “Down but Not Out: Vasectomy Is Faring Poorly Almost Everywhere—We Can Do Better to Make It a True Method Option”

**DOI:** 10.9745/GHSP-D-23-00240

**Published:** 2023-08-28

**Authors:** Roy Jacobstein, Scott Radloff, Farhad Khan, Kathryn Mimno, Manoj Pal, Jennifer Snell, Renae Stafford, Cheick Touré, Vandana Tripathi

**Affiliations:** a Consultant, Cary, NC, USA.; b The Bill & Melinda Gates Institute for Population and Reproductive Health, Johns Hopkins University, Baltimore, MD, USA.; c MOMENTUM Safe Surgery in Family Planning and Obstetrics, EngenderHealth, Washington, DC, USA.; d MOMENTUM Safe Surgery in Family Planning and Obstetrics, IntraHealth International, Chapel Hill, NC, USA.; e MOMENTUM Safe Surgery in Family Planning and Obstetrics, EngenderHealth, New Delhi, India.; f MOMENTUM Safe Surgery in Family Planning and Obstetrics, IntraHealth International, Bamako, Mali.

See related articles by Labrecque and Jacobstein et al.

We appreciate Labrecque’s praise of our analysis of worldwide vasectomy use and his endorsement of our programmatic recommendations.^1^ He points out that the vasectomy prevalence estimates for Canada in the 2022 United Nations compendium of surveys^2^ that we drew upon for our study are inconsistent, provides supplemental data on vasectomy use in Canada, and speculates that other countries may have “similar flawed estimates.” He also questions the “validity of using surveys performed more than 10 years ago . . . for estimating 2022 prevalence.” We address each point in turn.

Regarding Canada, we agree a decline in vasectomy prevalence of 14–15 percentage points in 4 years is highly unlikely. We welcome Labrecque’s provision of service data from Quebec and other provinces. These data confirm a decline in vasectomies performed, especially during the 2000s—but only at about one-third of the decline in prevalence reported in Canada’s last 2 surveys (22% in 2002; 7.4% in 2006). This level would place Canada with the world’s second-highest vasectomy prevalence after South Korea rather than the eighth highest vasectomy prevalence (among 197 countries and areas). We also point out that, importantly, there is no similar incongruity among the other 94 countries whose surveys we included in our analysis. That is, this Canadian anomaly does not represent a wider problem with the United Nations data compendium, as Labrecque’s letter suggests.

Regarding Labrecque’s 2 points in his final paragraph, it should be understood that our calculations of the number of worldwide vasectomy users were based not on our aggregation of estimates of individual country prevalence levels but rather on the United Nation’s own 2019 estimates of population totals and vasectomy prevalence in various regions, subregions, and developmental areas of the world.^3^ Also, regarding survey recency, most of the 95 countries in our analysis have a last survey report from the most recent decade before COVID-19-related interruptions in conducting surveys (2011–2020). This includes 16 of the 20 countries with the world’s highest vasectomy prevalence (Table 1 in our article). The 4 exceptions are Canada (previously discussed), Belgium (2010), Bhutan (2010), and South Korea (2009). These latter 2 Asian countries—first and third in the world in country rank on vasectomy prevalence—were included to show that some current or former low- and middle-income countries (LMICs) were, longer ago, already consistently “walking the talk” about broadening method choice and ensuring constructive male engagement in family planning, including having widespread availability and substantial use of vasectomy. Similarly, in Latin America, Costa Rica has had consistently high vasectomy prevalence, above 5% since 2010, and Colombia doubled its vasectomy prevalence to almost 4% by 2016.

Finally, as Labrecque implies, due to very large populations of women of reproductive age in China and India, small variations in estimates of vasectomy prevalence can have substantial impact on estimates of the number of vasectomy users there and globally. However, we have no reason to believe either country’s estimates of vasectomy prevalence are overestimated or underestimated. Furthermore, even if their prevalence estimations were “low,” and there were several million or more vasectomy users in either country, our article’s main points would remain valid and noteworthy.

- Despite the world’s population having more than doubled, desired family size having fallen in almost all countries, and modern contraceptive use having risen markedly, **vasectomy use is surprisingly low and declining—barely half of what it was 4 decades ago, in 1982**.
- Only 7 countries in the world, including 3 LMICs, registered increases in vasectomy prevalence during a most recent decade.
- **In almost all LMICs, vasectomy use remains negligible, with prevalence of 0.1% or less**. And even in many higher-income countries, vasectomy prevalence is low and/or declining.
- Countries with the lowest gender inequality are among those with the highest vasectomy prevalence and vice versa.
- **Female to male disparities in permanent method use have widened globally over the past 3 decades**, from 3:1 in 1990 to 13:1 in 2019, and in some regions and countries, the female to male disparity is much greater (e.g., 76:1 in India).

Surely, we can do better to ensure that this highly effective male method is more widely and accurately understood and more equitably available and accessible.

## Notes

**Peer Reviewed**

**First published online:** July 11, 2023.
